# Nutritive Value Variation and In Vitro Digestibility of Hempseed Meal

**DOI:** 10.3390/ani11123481

**Published:** 2021-12-07

**Authors:** Kristen June Jacobson, Lea Ann Kinman, Walter Franklin Owsley, James Pierre Muir, William Brandon Smith

**Affiliations:** 1 Department of Animal Science, Tarleton State University, Stephenville, TX 76401, USA; sw_jacobson@tarleton.edu (K.J.J.); kinman@tarleton.edu (L.A.K.); owsley@tarleton.edu (W.F.O.); 2 Texas A&M AgriLife Research, Stephenville, TX 76401, USA; jim.muir@ag.tamu.edu; 3 Department of Wildlife and Natural Resources, Tarleton State University, Stephenville, TX 76401, USA

**Keywords:** *Cannabis sativa*, hemp, hempseed meal, nutritive value, variability, ruminants

## Abstract

**Simple Summary:**

While there have been international studies on hemp as a livestock feed source, information is limited in the U.S.A. Hempseed meal, the byproduct of oil production, is relatively unexplored. Our research focused on determining variability among hemp seed meal sources and batches within sources, through chemical analysis and *in vitro* digestibility of samples. Our research found nutritive value variability among batches, but not sources, of hempseed meal. *In vitro* digestibility only slightly decreased when hempseed meal was included at increasing percentages as a protein replacement in the ration. These data indicate that hempseed meal may be an effective source of crude protein for inclusion in ruminant livestock rations.

**Abstract:**

Hempseed meal (HSM) is left after oil extraction of hemp and may act as a protein source in livestock. The first phase of this research evaluated variation in nutritive value and *in vitro* dry matter digestibility (IVDMD) of HSM from various sources in North America; the second phase utilized IVDMD to evaluate the efficacy of hempseed meal as an ingredient in ruminant feed. In phase one, the source had no contribution to variance for neutral detergent fiber (NDF), acid detergent fiber (ADF), acid detergent lignin (ADL), or crude protein (CP) (*p* ≥ 0.20). However, batch within source contributed to variation for NDF (50%), ADF (37%), ADL (13%), and CP (31%; *p* ≤ 0.01). Irrespective of differences in nutritive value, there was no contribution to variation (*p* = 0.23) of any measured response on *in vitro* true digestibility (53.0%). In phase two, two experiments evaluated HSM IVDMD as (1) a concentrate replacement or (2) a protein replacement in rations at varying rates. In the first experiment, IVDMD decreased (*p* < 0.05) with increasing levels of HSM. In the second experiment, IVDMD decreased (*p* < 0.01) as HSM inclusion increased. Although IVDMD decreased as HSM inclusion increased, values still met the digestibility threshold for ruminant rations, indicating that HSM has potential as an alternative protein ingredient.

## 1. Introduction

Recent US 2018 Farm Bill hemp legislation may open the way for an alternative livestock feed protein source. Hemp and marijuana are both derived from the plant *Cannabis sativa* L., with the sole difference that hemp must contain less than 0.3% tetrahydrocannabinol (THC), the psychoactive component in the plant [1]. Over time, marijuana and hemp have been selectively bred to contain differing cannabidiol (CBD) and THC levels. While there is genetic differentiation between the two, both have similar variation and are no more genetically distant from one another than from other strains within their subgroup [2].

Hemp oil, derived from *C. sativa* seed, has gained popularity over recent years for its use in cosmetics, pharmaceuticals, and human food products [3]. When oil is extracted from hemp seeds through either chemical or mechanical methods, a high-protein hempseed meal (HSM) remains [4]. Hempseed meal composition is dependent on plant and seed quality prior to oil extraction, which can be affected by weather, location of growth, and variation in plants, among other factors [3]. The crude protein content of HSM ranges between 316 and 356 g kg^−1^ [4,5,6,7,8]. Data on fiber content are limited, with NDF values of 372 to 507.9 g kg^−1^ [4,5,6,7]. 

North American hemp industry growth as a result of the 2018 Farm Bill may prove beneficial to the livestock industry. Alternative feed sources are continually being evaluated to advance production efficiency and profitability. While there have been studies on hemp as a livestock feed source, information is limited, especially on U.S.-grown hemp. As of the time of this publication, HSM has not been approved as a feed ingredient by either the Food and Drug Administration or the Association of American Feed Control Officials. Our objectives were to evaluate variation in nutritive value and *in vitro* dry matter digestibility (IVDMD) among commercial hempseed meal sources and batches, and to determine the potential of hempseed meal as a feed ingredient for ruminant rations.

## 2. Materials and Methods

### 2.1. Sample Collection

Hemp processing facilities within the United States of America that used cold pressing as the method of oil extraction were contacted to obtain HSM samples. Processors were asked to provide distinct samples from as many batches as possible. All hemp seed was collected from *C. sativa* plants containing less than 0.3% THC per the 2018 Farm Bill [1]. 

### 2.2. Variability in Nutritive Value

Upon receipt, HSM samples were dried in a forced-air oven at 55 °C until weight loss ceased. Samples were then ground to pass through a 2-mm screen in a Wiley mill (Arthur H. Thomas Co., Philadelphia, PA, USA); a subsample was ground to pass through a 1-mm screen. 

The experimental design for determining the source of variation was completely randomized. A total of four processing facilities and 15 hempseed meal batches were represented in the experiment. Four laboratory replicates of each batch × source combination were assayed. 

Carbon and nitrogen concentrations were determined by combustion using the Dumas total combustion method in a Leco Cornerstone CN 828 (Elementar Americas, Mt. Laurel, NJ, USA; Method 990.09) [9]. Nitrogen concentration was used to calculate crude protein (CP) content by multiplying by 6.25 [10]. Neutral detergent fiber (NDF), acid detergent fiber (ADF), and acid detergent lignin (ADL) were measured with the ANKOM^200^ Fiber Analyzer (ANKOM Technology Corporation, Fairport, NY, USA) using modified methods [11] originally described by [12]. Acid detergent lignin was measured by the sulfuric acid method (Method 973.18) [9] An ANKOM Daisy^II^ incubator (ANKOM Technology Corporation, Fairport, NY, USA) was used to determine *in vitro* true digestibility (IVTD) using a modification [11] of the *in sacco* disappearance method originally described by [13]. Rumen fluid was collected from a ruminally-fistulated steer at the Texas A&M AgriLife Research and Extension Center in Stephenville TX. The steer was offered *ad libitum* access to ‘Coastal’ bermudagrass (*Cynodon dactylon* [L.] Pers.) hay with a minimum 120 g CP kg^−1^ DM.

### 2.3. In Vitro Dry Matter Digestibility Experiments

The experimental design for each of the experiments in which the effect of HSM inclusion on IVDMD was evaluated was a generalized complete block design. Block was designated as a single rumen fluid collection, and each experiment included two blocks. Each dietary treatment was replicated three times within each block, with the flask serving as the experimental unit.

The HSM used in mixtures for the *in vitro* experiments was a composite of the 15 batches collected from four oil processing factories (336 g CP kg^−1^ DM). Steam flaked corn (*Zea mays* L.), bermudagrass hay, alfalfa (*Medicago sativa* L.) pellets, and soybean (*Glycine max* L.) meal were purchased at a local feed store. All ingredients were ground to pass through a 2-mm screen in a Wiley mill (Arthur H. Thomas Co., Philadelphia, PA, USA). In the first experiment, HSM replaced the concentrate (steam-flaked corn) in a 60:40 forage-to-concentrate ratio mixture at 0, 250, 500, 750, and 1000 g kg^−1^. The forage in this mixture was bermudagrass hay. In the second experiment, isonitrogenous (book value) mixtures were formulated in which HSM replaced soybean meal and alfalfa at 0, 250, 500, and 750 g kg^−1^. The complete composition of feeds tested in both experiments are presented in Table 1.

Modified methods of the procedure originally described by [14] were utilized. For each experiment, 0.5 g samples were weighed into 125 mL Erlenmeyer flasks with 40 mL of a 1:5 ratio of buffer solution B and buffer solution A [15]. Ten mL of CO_2_-flushed rumen fluid collected from a ruminally-fistulated steer was then added to each flask. Flasks were sealed with a rubber stopper fitted with an airlock, placed in a Fisherbrand Isotemp Shaking Water Bath (Thermo Fisher Scientific, Newington, NH, USA) at 39 °C, and agitated at 30 RPM for 48 h. Then, 2 mL HCl and 0.5 g pepsin were added to each flask, and stoppers were removed. After 48 h, samples were filtered through P8 filter paper and dried for 2 h at 105 °C.

### 2.4. Statistical Analysis

Data were analyzed using SAS v. 9.4 (SAS Institute, Inc., Cary, NC, USA). Prior to analysis, raw data were tested using the NORMAL option of PROC UNIVARIATE to ensure data normality. Normality was assumed when Shapiro-Wilk’s *W* met or exceeded 0.9 [16,17]. 

For the experiment evaluating the source of variation in nutritive value, response variables were analyzed using the linear mixed model procedure (PROC MIXED) in SAS using the COVTEST option for random-effects models. Random effects included source (processor), batch within source, and replicate within batch by source (replicate was understood to represent the laboratory replicate and not a statistical replicate). 

For the IVDMD experiments, response variables were analyzed using the generalized linear mixed models procedure (PROC GLIMMIX) in SAS. The fixed effect was treatment, and the random effects were block and treatment by block. Orthogonal polynomial contrasts were tested for linear and quadratic effects of HSM inclusion. Coefficients for contrasts were determined using PROC IML. In experiment 1, linear coefficients were −0.63, −0.32, 0, 0.32, and 0.63 for 0, 250, 500, 750, and 1000 g HSM kg^−1^ feed mixture, respectively. Quadratic coefficients were 0.53, −0.27, −0.53, −0.27, and 0.53 for 0, 250, 500, 750, and 1000 g HSM kg^−1^ feed mixture, respectively. In experiment 2, linear coefficients were −0.67, −0.22, 0.22, and 0.67 for 0, 250, 500, and 750 g HSM CP kg^−1^ feed mixture CP, respectively. Quadratic coefficients were 0.5, −0.5, −0.5, and 0.5 for 0, 250, 500, and 750 g HSM CP kg^−1^ feed mixture CP, respectively.

For all experiments, denominator degrees of freedom were adjusted using the Kenward-Roger approximation method [18]. The α-level for mean differences was set at 0.05. When interactions had *p* < α, the interaction was discussed; otherwise, the main effects were discussed. Means separations were performed based on *F*-protected *t*-tests using Tukey-Kramer’s HSD.

## 3. Results

### 3.1. Variability in Nutritive Value

**Figure 1:**
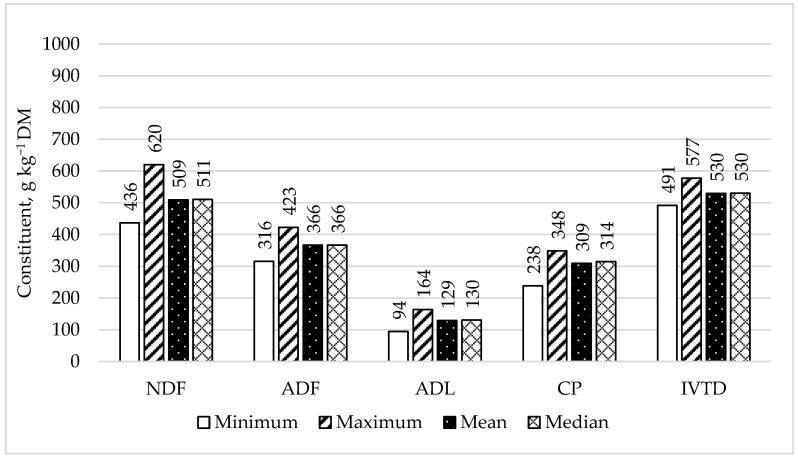
Minimum, maximum, mean, and median observations for neutral detergent fiber (NDF), acid detergent fiber (ADF), acid detergent lignin (ADL), crude protein (CP), and in vitro true digestibility (IVTD) of 15 hempseed meal samples collected from four oil processing facilities.

Mean, median, minimum, and maximum values of nutritive value of HSM are presented in Figure 1. Source had no contribution to variance for NDF, ADF, ADL, or CP (Table 2; *p* ≥ 0.20). However, batch within source contributed to variation for NDF (μ = 50.9%; *p* = 0.01), ADF (μ = 36.8%; *p* = 0.01), ADL (μ = 12.9%; *p* < 0.01), and CP (μ = 30.9%; *p* < 0.01). Regardless of the differences in nutritive value, there was no contribution to variation (*p* ≥ 0.23) of any measured effect on IVTD (μ = 53.0%). Therefore, while variation existed among HSM samples with respect to nutritive value, digestibility was not affected. 

### 3.2. In Vitro Dry Matter Digestibility

In the first experiment, IVDMD decreased linearly (*p* < 0.01) with increasing HSM inclusion (Table 3). Feed mixture IVDMD was greatest (*p* < 0.05) from 0 g HSM kg^−1^ feed (406 g kg^−1^) and least from 1000 (225 g kg^−1^), with 250, 500, and 750 intermediate (Table 3). 

In the second experiment, IVDMD, again, decreased linearly (*p* < 0.01) with HSM inclusion. However, this level of inclusion was based on dietary CP rather than absolute ratios of feed ingredients. Feed mixture IVDMD was greatest (*p* < 0.05) at 0 (771 g kg^−1^), followed by 250 and 500, and least from 750 (643 g kg^−1^; Table 4). 

## 4. Discussion

### 4.1. Variability in Nutritive Value

Table 5 shows the chemical analysis of HSM from various studies. Hempseed meal crude protein (CP) is comparable to that of other common protein feed sources. A study performed in Italy that examined 20 hemp genotypes from differing geographical locations all grown under the same conditions, found CP contents to range from 316 to 356 g kg^−1^ with an average of 337 g kg^−1^ [8]. In a Hemp Feed Coalition summary of certificates of analysis from 39 sources of American-grown and -processed HSM of three varieties from 10 states, the average CP content was 335 g kg^−1^ [19]. Other studies found similar CP values [4,5,6,7], and our present study was in agreement with these findings. 

Neutral detergent fiber (NDF) values from previous studies vary greatly as seen in Table 4 [4,5,6,7]. Acid detergent fiber (ADF) values also vary [4,5,7]. Acid detergent lignin (ADL) was only determined in one study [5].

#### Sources of Nutritive Value Variation

Temperature affects seed formation in hemp with ideal temperatures >27 °C [21]. Protein synthesis may increase when seed filling occurs during periods of high temperatures because of more efficient N transfer to the seeds [22]. This was supported by the findings of [23] in which seeds grown during a year with higher recorded temperatures had greater CP content than seeds grown the year prior with lower recorded temperatures. Therefore, the temperature may also have had an effect on variability in nutrient composition between HSM batches; however, this is not consistent with the lack of variability among sources in our study regardless of the differing geographical locations. 

Grieshop et al. [24] found that growing and processing conditions, as well as varietal differences, all contributed to the nutrient composition of SBM. Processing conditions, including temperature and time, can affect the nutrient composition of SBM.

### 4.2. In Vitro Dry Matter Digestibility

Our observations of IVDMD in the protein replacement mixture were comparable to those of an in vivo feed trial done on sheep where HSM replaced canola meal at 0, 250, 500, 750, and 1000 g kg^−1^, resulting in DM digestibility coefficients of 0.66, 0.63, 0.64, 0.61, and 0.64, respectively [5]. These data support our findings and encourage the applicability of HSM as a protein replacement in ruminant rations. Decreases in IVDMD when HSM replaced SBM/alfalfa were less than those observed when HSM replaced steam-flaked corn, but any conclusions on diet suitability should be made with caution without data from feeding experiments to determine effects on DMI and whether there are any anti-nutritional factors present. 

Changes in observed IVDMD could be due to a number of interactions of the nutrients in the tested mixtures. While fiber concentrations were not evaluated in experiment 1, Table 1 shows that total fiber (NDF) ranged by only 50 g kg^−1^, indicating that it is likely not the reason for observed differences. While digestibility values were unsuitable when HSM replaced the concentrate portion of the mixture in experiment 1, all digestibility values for mixtures with HSM as a protein replacement in experiment 2 had digestibility percentages acceptable for ruminant rations. Although there was a decrease in digestibility as HSM inclusion increased as a protein source in the mixture, the difference was slight, and dry matter digestibility remained sufficient to meet ruminant needs at all levels of inclusion. Further studies with a 100% HSM inclusion rate and replacement of protein sources more similar in chemical composition are warranted.

## 5. Conclusions

Our research found nutritive value variability among batches of HSM, which can be attributed to a variety of causes including genetics, environmental factors, and processing techniques. However, IVTD was not affected by this variability, and we found no variability among sources of HSM. Hempseed meal IVDMD only slightly decreased when HSM was included at increasing percentages as a protein replacement in the feed mixture, indicating it may be a potential source of supplemental protein in ruminant diets. Data from current publications in combination with the results of this research indicate there is promise for the inclusion of HSM in ruminant rations.

## Figures and Tables

**Table 1:** Composition (g kg^−1^) of feed mixtures tested in the *in vitro* experiments of hempseed meal digestibility.

	Experiment 1: Hempseed Meal Replacement of Concentrate, g kg^−1^					Experiment 2: Hempseed Meal Replacement of Dietary Crude Protein, g kg^−1^			
Feedstuff	0	250	500	750	1000	0	250	500	750
Steam-flaked corn	600	450	300	150	-	310	310	310	315
Bermudagrass hay	400	400	400	400	400	-	-	120	250
Alfalfa meal pellets	-	-	-	-	-	620	530	280	-
Soybean meal	-	-	-	-	-	70	15	-	-
Hempseed meal	-	150	300	450	600	-	145	290	435
Nutritive value									
NDF	-	-	-	-	-	295	284	326	340
ADF	-	-	-	-	-	213	210	220	207
ADL	-	-	-	-	-	50	59	59	53
CP	-	-	-	-	-	120	140	150	190

**Table 2:** Random effects estimates of neutral detergent fiber (NDF), acid detergent fiber (ADF), acid detergent lignin (ADL), crude protein (CP), and in vitro true digestibility (IVTD) from 15 hempseed meal samples collected from four oil processing facilities.

Effect	Estimate	SE ^1^	Z-Value	*p*-Value	Contribution to Variance ^2^
NDF					
Rep (source by batch)	0	-	-	-	-
Batch (source)	0.000969	0.000434	2.23	0.01	65.08
Source	0	-	-	-	-
Residual	0.000520	0.000134	3.87	< 0.01	34.92
ADF					
Rep (source by batch)	0	-	-	-	-
Batch (source)	0.000381	0.000172	2.21	0.01	44.82
Source	0.000375	0.000441	0.85	0.20	44.12
Residual	0.000094	0.000024	3.87	<0.01	11.06
ADL					
Rep (source by batch)	0	-	-	-	-
Batch (source)	0.000287	0.000121	2.38	<0.01	68.50
Source	0.000096	0.000143	0.67	0.25	22.91
Residual	0.000036	0.000009	3.87	<0.01	8.59
CP					
Rep (source by batch)	0	-	-	-	-
Batch (source)	0.000964	0.000371	2.60	<0.01	96.50
Source	0	-	-	-	-
Residual	0.000035	0.000013	2.74	<0.01	3.50
IVTD					
Rep (source by batch)	0	-	-	-	-
Batch (source)	0.000050	0000068	0.73	0.23	9.58
Source	0	-	-	-	-
Residual	0.000472	0.000100	4.74	<0.01	90.42

^1^ SE = standard error. ^2^ Contribution to variance is measured as the percent of the total variance accounted for by the measured effect.

**Table 3:** In vitro dry matter digestibility (IVDMD) of a 60:40 forage-to-concentrate ratio mixture in which hempseed meal replaced the concentrate portion at various levels.

	Hempseed Meal Replacement of Concentrate, g kg^−1^					Contrasts	
	0	250	500	750	1000	Linear	Quadratic
IVDMD, g kg^−1^ DM	406 ^a^	379 ^ab^	332 ^ab^	285 ^ab^	225 ^b^	<0.01	0.56

^a,b^ Different letters indicate significant differences in values (*p* < 0.05) according to Tukey’s honest significant difference.

**Table 4:** In vitro dry matter digestibility (IVDMD) of isonitrogenous rations in which hempseed meal replaced the crude protein portion at various levels.

	Hempseed Meal Replacement of Crude Protein, g kg^−1^				Contrasts	
	0	250	500	750	Linear	Quadratic
IVDMD, g kg^−1^ DM	771 ^a^	716 ^b^	693 ^b^	643 ^c^	<0.01	0.86

^a–c^ Different letters indicate significant differences in values (*p* < 0.05) according to Tukey’s honest significant difference.

**Table 5:** Nutritive value parameters of hempseed meal samples from published literature.

Cultivar	Extraction Method	CP ^1^	NDF ^2^	ADF ^3^	ADL ^4^	Reference
CAN19	Chemical	345	-	-	-	[8]
CAN20	Chemical	356	-	-	-	[8]
CAN24	Chemical	331	-	-	-	[8]
CAN 26	Chemical	345	-	-	-	[8]
CAN39	Chemical	320	-	-	-	[8]
CAN40	Chemical	354	-	-	-	[8]
CAN48	Chemical	339	-	-	-	[8]
CAN51	Chemical	336	-	-	-	[8]
CAN58	Chemical	346	-	-	-	[8]
Finola	Chemical	317	-	-	-	[8]
Carmagnola	Chemical	334	-	-	-	[8]
CS	Chemical	316	-	-	-	[8]
Fibranova	Chemical	325	-	-	-	[8]
Fedora	Chemical	339	-	-	-	[8]
Futura 75	Chemical	337	-	-	-	[8]
Felina 32	Chemical	343	-	-	-	[8]
Ferimon	Chemical	344	-	-	-	[8]
Codimono	Chemical	336	-	-	-	[8]
Carmaleonte	Chemical	345	-	-	-	[8]
Kc Dora	Chemical	332	-	-	-	[8]
Various	Cold-pressed	335	436	346	-	[19]
Finola	Cold-pressed	385	449	-	-	[20]
Finola	Cold-pressed	369	434	-	-	[20]
-	-	344	393	103	-	[7]
Finola	Cold-pressed	336	382	336	-	[4]
-	-	320.8	507.9	390.4	131.9	[5]
Carmagnola	Chemical	337	-	-	-	[21]
CS	Chemical	348	-	-	-	[21]
Fibranova	Chemical	351	-	-	-	[21]
Futura 75	Chemical	342	-	-	-	[21]
Felina 32	Chemical	351	-	-	-	[21]
Ferimon	Chemical	331	-	-	-	[21]
Unika-b	Cold-pressed	249	372	-	-	[6]

^1^ CP = crude protein, g kg^−1^ DM; ^2^ NDF = neutral detergent fiber, g kg^−1^ DM; ^3^ ADF = acid detergent fiber, g kg^−1^ DM; ^4^ ADL = acid detergent lignin, g kg^−1^ DM.

## Data Availability

The data presented in this study are available on request from the corresponding author. The data are not publicly available due to privacy concerns related to donated samples from commercial entities.
